# Management of dental care of patients on immunosuppressive drugs for chronic immune-related inflammatory diseases: a survey of French dentists’ practices

**DOI:** 10.1186/s12903-023-03258-7

**Published:** 2023-08-09

**Authors:** Alice Bourgoin, Kevimy Agossa, Raphaele Seror, Mathurin Fumery, Loredana Radoi, Marjolaine Gosset

**Affiliations:** 1 Service de Médecine Bucco-Dentaire, AP-HP, Hôpital Charles Foix, Ivry/Seine, F-94200 France; 2 Université de Lille, Inserm, CHU Lille, U1008, Lille, F-59000 France; 3 Department of Periodontology, Faculty of Dentistry, University of Lille, Place De Verdun, Lille, France; 4 Department of Rheumatology, Université Paris-Saclay, Assistance Publique-Hôpitaux de Paris (AP-HP), CHU Bicêtre, Le Kremlin-Bicêtre, France; 5 Department of Gastroenterology, Amiens University Hospital, Picardie University, Amiens, France; 6 Department of GastroenterologyPériTox Laboratory, Périnatalité & Risques Toxiques, UMR-I 01 INERIS, Picardie Jules Verne University, Amiens, France; 7 Service de Médecine Bucco-Dentaire, AP-HP, Hôpital Louis Mourier, Colombes, F-92700 France; 8 CESP, INSERM, Exposome and Heredity Group, Université Paris-Saclay, Villejuif, France; 9 Laboratoire d’Excellence INFLAMEX, Paris, France

**Keywords:** Biologic disease-modifying antirheumatic drugs, DMARDs, Immunosuppressants, Oral care, Invasive procedure

## Abstract

**Objectives:**

The aim of the study was to provide an overview of the practices of French general dentists (GDs) and specialists (SDs) concerning the management of patients with inflammatory bowel diseases (IBDs), rheumatic inflammatory diseases (IRDs), and vasculitis on biologic disease-modifying antirheumatic drugs (bDMARDs), conventional DMARDs, or immunosuppressants (ISs).

**Materials and methods:**

An online national cross-sectional survey with 53 questions was developed by a multidisciplinary team including rheumatologists, gastroenterologists and dentists based on their clinical experience. It was refined following a test with nine dentists in private practice and in hospital before being disseminated to the members of French scientific societies and colleges of dentistry teachers over 3 months. Responses of general dentists versus specialists were compared with respect to their experience in managing patients with IRDs or IBDs, knowledge/training, type of invasive procedure performed, management of medical treatment, perioperative oral-care protocols, and frequency of postoperative complications after invasive dental care procedures.

**Result:**

In total, 105 practitioners fully completed the survey (participation rate 11.1%). SDs more frequently performed invasive surgical procedures and were more aware of the recommendations of learned societies than GDs. They encountered more post-operative complications for patients on bDMARDs. For both SDs and GDs, most patients were managed without stopping treatment and pre- and postoperative antibiotics were prescribed to more than 75% of patients. When medical treatment was stopped, the decision was made by the prescribing physician.

**Conclusion:**

Complications were reported more frequently by SDs when highly invasive procedures were performed on patients under active drug therapy. Certain common procedures, such as scaling and root planing, appear to be safe, regardless of treatment management. However, adapted guidelines for the practice of dentistry are needed to standardize the management of patients on bDMARDS, conventional DMARDs, or ISs.

**Clinical relevance:**

French dentists perform a wide range of oral procedures on patients on bDMARDS, conventional DMARDs, or ISs under antibiotic coverage and antiseptic mouthwashes. SDs reported more postoperative complications after extensive invasive procedures for patients under active drug therapy, despite their greater knowledge of recommendations on how to manage such patients.

**Supplementary Information:**

The online version contains supplementary material available at 10.1186/s12903-023-03258-7.

## Introduction

There is increasing evidence of a bidirectional relationship between oral and general health, underscoring the importance of treating oral diseases prior to the initiation of certain systemic therapies [1]. Collaboration between dentists and physicians is necessary for oral care, as described in transdisciplinary consensus articles for patients with diabetes mellitus or cardiovascular diseases [2, 3]. Such an approach is also necessary for patients with immune-related inflammatory disorders (IMIDs), such as chronic inflammatory bowel diseases (IBDs) and chronic inflammatory rheumatic diseases (IRDs), particularly when treated with immunosuppressants (ISs), conventional disease-modifying antirheumatic drugs (DMARDs), and/or biologics (bDMARDs). Different populations show considerable variation in the frequency of the disease, but the estimated prevalence of IMIDs in Western society is 5 to 7% [4]. The development of effective biologics over the past few decades has dramatically improved clinical outcomes and new molecules are emerging regularly [5]. Thus, it is important for dentists and prescribing physicians to be able to refer to common recommendations when performing oral care on these patients. However, to date, there are no international transdisciplinary recommendations on the management of invasive oral procedures in patients with IMIDs on ISs, DMARDs, or bDMARDs.

To date, in France, three scientific societies have proposed national recommendations: the French Society of Oral Surgery (SFCO) [6], the Club of Chronic Inflammatory Rheumatism (CRI), part of the French Society of Rheumatology [7], and the National Agency for Drug Safety (ANSM) [8]. They agree on the elimination of infectious foci and the treatment of caries and periodontal disease by general dentists (GDs) or specialized dentists (SDs) working in a community-based dental office or hospital, before starting medical treatment [5, 9]. The challenge is to manage the risk of infection in oral care once the treatment has started. Indeed, these three guidelines diverge, rely on a low level of evidence, are not based on literature in the field of dentistry, provide no details by type of dental procedure, and have not been updated for more than 10 years for the ANSM and the SFCO [10]. This situation is confusing. There is therefore an urgent need to evaluate the knowledge and practices of dentists regarding invasive oral treatments in real-life situations. For this purpose, the existence of different training courses in dentistry and/ or oral care in France (supplemental Fig. 1) must be considered when assessing the knowledge and skills of dentists in the management of these patients.

Our objective of this study was to evaluate, through a national survey, the practices and knowledge of dentists with general or specialized practice concerning the risks and their prevention during the dental care of IMIDs patients on biologics or ISs in France.

## Methods

A multidisciplinary collaboration was established between research teams working in various fields, namely rheumatology (RS), gastroenterology (MF), oral surgery, oral implantology, and periodontology (AB, KA, LR, MG), to conduct a national cross-sectional survey among members of dental scientific societies concerning the dental management of patients with IMIDs, in particular, those with IRDs, IBDs, or vasculitis, receiving bDMARDS, DMARDs, and/or ISs. The dentists (KA, LR, MG) involved in this study all have an academic position and a hospital practice.

### Study population

In France, all dentists with an academic position are members of the National College of Academic Teachers (*College National d’Enseignants*—CNE) in periodontology (CNEP), oral surgery (CNECO), pediatric dentistry (CNEOP), or restorative dentistry and endodontics (CNEOC). They are simultaneously members of scientific societies such as the French Society of Oral Surgery (SFCO), the French Society of Periodontology and Oral Implantology (SFPIO), the French Society of Pediatric Dentistry (SFOP), and/or the French Society of Endodontics (SFE). Other members of these societies are specialist and general French dentists with a particular interest in a certain field of dentistry. In this study, all dentists reporting an activity limited to a field of specialization (oral surgery, periodontology, oral implantology, others) were considered to be “specialist dentists”. Thus, the distinction was not made based on the level of the degree declared.

As patients with IRDs and IBDs may be followed by non-specialized or specialized dentists in office-based or hospital-based dental services, we invited the participation of the members of these scientific societies and National Colleges of Academic Teachers in dentistry by email, which allowed us to reach a large panel of dentists. Therefore, members of the SFCO, SFPIO, SFE, SFOP, CNEP, CNECO, CNEOP, and CNEOC were invited to participate in the online survey via mailing lists.

Insofar as the scientific societies have members based in Europe or Africa, and as we wished to evaluate professional practices with reference to French recommendations (SFCO, CRI, and ANSM), the study was limited to members practicing in France. As many dentists are members of both colleges of academic teachers and scientific societies, it was not possible to accurately provide the number of dentists approached for the survey. According to the number of members declared in 2020 by the different scientific societies and academic colleges, it was estimated in consultation with these societies that about 2200 dentists (GP and SP) were invited to participate.

### Data collection

A semi-structured questionnaire containing 53 questions was developed by the authors (KA, RS, MF, LR, and MG), expert clinicians in the field, to assess the practices of professionals in real-life situation through this survey. The questionnaire was divided into five sections, of which the main points were:

1. the practitioner’s profile: gender, time since graduation, type of practice facility, and type of practice (generalist or specialist, with a choice of field of specialization), type of degree, and experience with patients with IRD or IBD (number of patients treated per month by type of disease, i.e., IBDs, joint diseases, or vasculitis, reason for the first visit, and patient’s background treatment)
2. the participant’s knowledge/training concerning the dental care of patients on biologics: yes/no, source of information, expected risks after dental invasive procedures
3. the management of oral care of patients on biologics: nature of invasive procedures performed, management of the medical treatment, biological tests prescribed preoperatively, protocol for the use of antiseptics and antibiotics, declaration of postoperative complications occurring after invasive dental care procedures, and the nature of complications
4. same as 2 but on conventional DMARDs and ISs
5. same as 3 but on conventional DMARDs and ISs.

The biologics listed were the following (the main trade names were provided): anti-TNFα, anti-IL-1β, anti-IL-6, anti-IL-12/23, anti-lymphocyte B, anti-lymphocyte T, anti-IL-17, anti-integrin, and the targeted synthetic DMARD anti-JAK. The ISs listed, with examples of trade names, were the following: azathioprine, methotrexate, and cyclophosphamide. Corticoids were also listed. Participants were given the possibility to fill in “other molecules” in a free field.

The questionnaire was tested by nine generalized or specialized dentists with a community-based private or hospital practice. Their feedback on the comprehension of the questions and the ease of answering them helped with improvement and finalization of the questionnaire. The questionnaire was distributed on a secure platform provided by the University of Paris (Lime Survey®), allowing online completion, storage of the answers, and data extraction. Participants were invited to complete the questionnaire by email in September 2020, with four reminders up to December 31, 2020. The email included a synopsis of the survey and the link to the online questionnaire.

Ethical approval for the study was granted by the APHP Centre Research Ethics Committee (“Comité d’éthique pour la recherche APHP centre”) on June 15, 2020, and is registered under IRB registration number: #00011928.

### Statistical analysis

Qualitative variables are presented as numbers and percentages and quantitative variables as numbers, means, and standard deviations.

We compared general dentists versus specialists with respect to their experience in managing patients with IRD or IBD (type of disease, number of patients, type of biologic/IS treatment), knowledge/training concerning dental care of patients with IRD or IBD, type of invasive procedure performed, management of medical treatment, perioperative oral-care protocols, and frequency of postoperative complications after invasive dental care procedures. Categorical variables were compared using Chi-squared or Fisher tests, as appropriate. Continuous endpoints were compared using Student’s t test.

Statistical analyses were performed using STATA software (Stata Statistical Software: Release 15. College Station, TX: StataCorp LLC). All tests were two-sided and a *p* value < 0.05 was the threshold for statistical significance.

## Results

### Study population

Among the 2200 dentists solicited to participate in the survey, 246 practitioners responded (participation rate of approximately 11.18%), but only 46.75% fully completed the questionnaire. This population was quite evenly distributed by sex, time since graduation, type of healthcare facility of practice, and type of activity (specialized or general dentists) (Table 1). Among the specialized dentists, almost half were involved in oral surgery and implantology. Finally, the majority of dentists (79.13%) were settled in regions of France other than Paris and its surrounds.

**Table 1:** Main characteristics of the responding dentists

	**All participants** ***N* = 115 *(%)***
**Gender**	
Male	50 *(43.47)*
Female	65 *(56.52)*
**Time since graduation (years)**	
< 10	48 *(42.00)*
10–20	37 *(32.00)*
> 20	30 *(26.00)*
**Type of healthcare facility of practice**	
Hospital exclusively (public or university)	41 *(36.00)*
Private office or private practice exclusively	39 *(34.00)*
Mixed (hospital and private practice)	35 *(30.00)*
**Type of activity**	
General dentist	61 *(53.00)*
Specialized dentist	54 *(47.00)*
Oral surgery and implantology	24 *(21.00)*
Periodontology and implantology	18 *(16.00)*
Others (endodontics, pediatric dentistry)	12 *(10.00)*
**Level of education**	
Dental surgeon degree	64 *(55.65)*
Specialty degree in oral surgery or oral medicine	35 *(30.43)*
Other post-graduate degrees	16 *(13.91)*
**Region of practice**	
Paris and Ile de France region	24 *(20.87)*
Other French regions	91 *(79.13)*

### Experience of French dentist in the management of oral care of patients on bDMARDs, conventional DMARDs, or ISs

In terms of the participants’ experience, practitioners primarily treated patients with IRD (91.3%) or IBD (87.83%). Regardless of the disease, the average number of patients who presented was less than 2 per month, and patients were mostly treated with anti-TNFα, corticosteroids, or methotrexate (Table 2). The most frequent oral procedures performed were tooth extraction(s) (> 84%) and scaling and root planing (> 72%) (Table 3). Finally, SDs performed more invasive and complex procedures (i.e. pre-implant, plastic or osseous surgery), whereas GDs performed mainly conservative care (e.g. dental pulp treatment, scaling, and root planing) (*p* < 0.05) (Table 3).

**Table 2:** Information on the participants’ experience in the management of patients with chronic immune-mediated inflammatory diseases

	**All participants**	**Specialized dentist activity**^**a**^	**General dentist activity**	***P-value***^**b**^
	*N* = 115 *(%)*	*N* = 54 *(%)*	*N* = 61 *(%)*	
**Patients with Chronic Rheumatoid Inflammatory Disease (IRDs)**				
Yes	105 *(91.30)*	48 *(88.89)*	57 *(93.40)*	0.38
No	10 *(8.70)*	6 *(11.11)*	4 *(6.50)*	
**Patients with Inflammatory Bowel Diseases (IBDs)**				
Yes	101 *(87.83)*	46 *(85.20)*	55 *(90.16)*	0.41
No	14 *(12.17)*	8 *(14.80)*	6 *(9.84)*	
**Patients with Vasculitis**				
Yes	72 *(62.61)*	37 *(68.20)*	35 *(57.38)*	0.21
No	43 *(37.39)*	17 *(32.80)*	26 *(42.62)*	
**Mean number of patients treated per month *(SD)***				
With IRDs	1.51 *(1.61)*	1.93 *(1.73)*	1.16 *(1.42)*	0.99
With IBDs	0.84 *(1.09)*	1.10 *(1.31)*	0.63 *(0.81)*	0.98
With vasculitis	0.39 *(0.74)*	0.57 *(0.98)*	0.20 *(0.24)*	0.98
**Type of biologics used by patients under care**^**c**^				
Anti-TNF	75 *(70.09)*	35 *(71.42)*	40 *(68.96)*	0.28
Anti-IL1	14 *(13.08)*	9 *(18.36)*	5 *(8.62)*	0.16
Anti-IL6	22 *(20.56)*	13 *(26.53)*	9 *(15.51)*	0.19
Anti-IL12/23	10 *(9.34)*	6 *(12.24)*	4 *(6.89)*	0.39
Anti-B cells	36 *(33.64)*	22 *(44.89)*	14 *(24.13)*	**0.02**
Anti-T cells	15 *(14.01)*	10 *(20.40)*	5 *(8.62)*	0.09
Anti-IL17	4 *(3.73)*	2 *(4.08)*	2 *(3.44)*	0.90
Anti-integrin	9 *(8.44)*	7 *(14.28)*	2 *(3.44)*	0.05
Anti-Jak	7 *(6.62)*	5 *(10.20)*	2 *(3.44)*	0.18
Did not know the name of the drug	31 *(28.97)*	13 *(26.53)*	18 *(31.03)*	0.60
**Type of immunosuppressants used by patients under care**^**d**^				
Corticosteroids	91 *(85.04)*	41 *(83.67)*	50 *(86.20)*	0.81
Azathioprine	45 *(42.05)*	24 *(48.97)*	21 *(36.20)*	0.14
Methotrexate	85 *(79.43)*	40 *(81.63)*	45 *(77.58)*	0.37
Cyclophosphamide	22 *(20.56)*	13 *(26.53)*	9 *(15.51)*	0.14
Did not know the name of the drug	10 *(9.34)*	5 *(10.20)*	5 *(8.62)*	0.77

*Abbreviation*: *SD* Standard deviation

^a^Specialized dentist activity: oral surgery, periodontology, endodontics, pediatric dentistry

^b^Pearson’s chi square test or Fisher’s exact test for categorical variables or Student’s test for continuous variables

^c^Eight practitioners who did not treat patients with IRD/IBD/vasculitis were excluded from the statistical analysis

^d^Eight practitioners who did not treat patients with IRD/IBD/vasculitis were excluded from the statistical analysis

**Table 3:** Information on the oral care performed by participants on patients taking biologics or immunosuppressants

**Type of oral procedures performed by the practitioners themselves**	**All practitioners**^**a**^ ***N* = 104 (%)**	**Specialized dentist activity**^**c**^ ***N* = 47 (%)**	**General dentist activity** ***N* = 57 (%)**	***P-value***	**All practitioners**^**b**^ ***N* = 103 (%)**	**Specialized dentist activity**^**c**^ ***N* = 47 (%)**	**General dentist activity** ***N* = 56 (%)**	***P-value***
	**In patients under biologics**				**In patients under immunosuppressants**			
None	1 *(0.96)*	1 *(2.13)*	0 *(0.00)*	0.26	2 *(1.94)*	0 *(0.00)*	2 *(3.57)*	0.19
Rubber dam application	38 *(36.54)*	6 *(12.77)*	32 *(56.14)*	**< 0.001**	40 *(38.83)*	6 *(12.77)*	34 *(60.71)*	**< 0.001**
Dental pulp treatment	43 *(41.35)*	4 *(8.51)*	39 *(68.42)*	**< 0.001**	43 *(41.74)*	4 *(8.51)*	39 *(69.64)*	**< 0.001**
Scaling and root planing	75 *(72.12)*	25 *(53.19)*	50 *(87.72)*	**< 0.001**	76 *(73.79)*	27 *(57.45)*	49 *(87.50)*	**< 0.001**
Dental extraction(s)	89 *(85.58)*	38 *(80.85)*	51 *(89.47)*	0.31	87 *(84.47)*	38 *(80.85)*	49 *(87.50)*	0.22
Osseous surgery	43 *(41.35)*	32 *(68.09)*	11 *(19.30)*	**< 0.001**	39 *(37.86)*	26 *(55.32)*	13 *(23.21)*	**0.001**
Mucosal or plastic surgery	28 *(26.92)*	20 *(42.55)*	8 *(14.04)*	**0.01**	27 *(26.21)*	19 *(40.42)*	8 *(14.29)*	**0.003**
Preimplant surgery	9* (8.65)*	9 *(19.15)*	0 *(0.00)*	**< 0.001**	9 *(8.74)*	7 *(14.89)*	2 *(3.57)*	**0.04**
Implant(s) placement	18 *(17.31)*	13 *(27.66)*	5 *(8.77)*	**0.01**	14 *(13.59)*	9 *(19.15)*	5 *(8.93)*	0.13

^a^Only practitioners who responded that they are concerned about the care of patients taking biologics (*n* = 104) were included in this analysis

^b^Only practitioners who responded that they were concerned about the care of patients taking immunosuppressants (*n* = 103) were included in this analysis

^c^Specialized dentist activity: oral surgery, periodontology, endodontics, pediatric dentistry

### Dentist training and knowledge of the professional recommendations

SDs most often reported to have been trained and to have knowledge on the management of patients on bDMARDs, conventional DMARDs, or ISs (*p* < 0.05). Their main sources of information were journal articles, scientific societies, and professional meetings (*p* < 0.05). They were more aware of the professional recommendations, notably for the bDMARDs. They declared the French Society of Oral Surgery (SFCO) as the main source of information (> 79.31%). The vast majority of practitioners who had no training or information expressed their willingness to gain some (> 97.87%) (Table 4).

**Table 4:** Information on participants’ training and knowledge in the management of patients on biologics or immunosuppressants

	**All practitioners** ***N* = 115 (%)**	**Specialized dentist activity**^**d**^ ***N* = 54 (%)**	**General dentist activity** ***N* = 61 (%)**	***P-value***	**All practitioners** ***N* = 115 (%)**	**Specialized dentist activity**^**d**^ ***N* = 54 (%)**	**General dentist activity** ***N* = 61 (%)**	***P-value***
	**In patient under biologics**				**In patients under immunosuppressants**			
**Training/knowledge about oral care**								
No	48 *(41.74)*	17 *(31.48)*	31 *(50.82)*		47 *(40.87)*	17 *(31.48)*	30 *(49.18)*	
Yes	67 *(58.26)*	37 *(68.52)*	30 *(49.18)*	**0.03**	68 *(59.13)*	37 *(68.52)*	31* (50.82)*	0.05
**Source of training/information**^**a**^								
Scientific societies	40 *(59.70)*	25 *(67.57)*	15 *(50.00)*	0.14	44 *(64.71)*	25 *(67.57)*	19 *(61.29)*	0.59
Journal articles	48 *(71.64)*	28 *(75.68)*	20 *(66.67)*	0.41	49 *(72.06)*	28 *(75.68)*	21 *(67.74)*	0.46
Congresses	26 *(38.81)*	20 *(54.05)*	6 *(20.00)*	**0.004**	25 *(36.76)*	18 *(48.65)*	7 *(22.58)*	**0.02**
Pharmaceutical laboratories	1 *(1.49)*	1 *(2.70)*	0 *(0.00)*	0.36	2 *(2.94)*	2 *(5.40)*	0 *(0.00)*	0.18
Undergraduate and post-graduate training and others	12 *(17.91)*	5 *(13.51)*	7 *(23.33)*	0.29	14 *(20.58)*	9* (24.32)*	5 *(16.12)*	0.37
**Feeling of not being sufficiently trained or informed**^**a**^								
No	42 *(62.69)*	22* (59.46)*	20 *(66.67)*		37 *(54.42)*	19 *(51.35)*	18* (58.06)*	
Yes	25 *(37.31)*	15 *(40.54)*	10 *(33.33)*	0.54	31 *(45.59)*	18 *(48.65)*	13 *(41.94)*	0.58
**Wish to have training about oral care**^**b**^								
No	1 *(2.08)*	0 *(0.00)*	1 *(3.23)*		1 *(2.13)*	0 *(0.00)*	1 *(3.33)*	
Yes	47 *(97.92)*	17 *(100.00)*	30 *(96.77)*	0.45	46 *(97.87)*	17 *(100.00)*	29 *(96.67)*	0.44
**Knowledge of the professional recommendations on oral care**^**a**^								
No	59 *(51.30)*	21 *(38.89)*	38 *(62.30)*		57 *(49.57)*	22 *(40.74)*	35 *(57.38)*	
Yes	56 *(48.7)*	33 *(61.11)*	23* (37.70)*	**0.01**	58 *(50.43)*	32 *(59.26)*	26 *(42.62)*	0.07
**Knowledge of the professional recommendations from**^**c**^								
***Club Rhumatisms & Inflammation (CRI)***								
No	41 *(73.21)*	19 *(57.58)*	22 *(95.65)*		46 *(79.31)*	20 *(62.50)*	26 *(100.00)*	
Yes	15 *(26.79)*	14 *(42.42)*	1 *(4.35)*	**0.002**	12* (20.69)*	12 *(37.50)*	0 *(0.00)*	**< 0.001**
***National Drug Regulatory Authority (ANSM)***								
No	38 *(67.86)*	23 *(69.70)*	15 *(65.22)*		35 *(60.34)*	22 (68.75)	13 *(50.00)*	
Yes	18 *(32.14)*	10* (30.30)*	8 *(34.78)*	0.72	23 *(39.66)*	10 *(31.25)*	13 *(50.00)*	0.14
***French Society of Oral Surgery (SFCO)***								
No	10 *(17.86)*	6 *(18.18)*	4 *(17.39)*		12 *(20.69)*	6 *(18.75)*	6 *(23.08)*	
Yes	46 *(82.14)*	27 *(81.82)*	19 *(82.61)*	0.93	46 *(79.31)*	26 *(81.25)*	20 *(76.92)*	0.68
**Implementation of recommendations in the personal practice**^**c**^								
No	1 *(1.79)*	0 *(0.00)*	1 *(4.35)*		0* (0.00)*	0 *(0.00)*	0 *(0.00)*	
Yes	55 *(98.21)*	33 *(100.00)*	22 *(95.65)*	0.22	58 *(100.00)*	32* (100.00)*	26 *(100.00)*	--

^a^Only practitioners who responded ‘yes’ to the question about training/information

^b^Only practitioners who responded ‘no’ to the question about training/information

^c^Only practitioners who responded ‘yes’ to the question about knowledge of the professional recommendations

^d^Specialized dentist activity: oral surgery, periodontology, endodontics, pediatric dentistry

### Management of oral care

The main risks expected by practitioners were infections (≥ 98.13%) and delayed healing (≥ 84.11%) (Supplemental Table 1). Most practitioners did not prescribe biological tests before performing oral procedures (> 71.84%) but considered discontinuing the drug in consultation with the prescribing physician (> 76.7%). Antiseptic mouthwashes were frequently prescribed before and for long-term use after invasive oral procedures, whereas antibiotics were mainly prescribed afterwards, and until mucosal healing (Supplemental Table 1).

### Post-operative complications after invasive oral procedures declared by GDs and SDs

Most practitioners (> 80%) reported no postoperative complications after oral procedures (Table 5). Complications were mainly associated with tooth extraction (< 18.45%), and were more frequently reported by SD (*p* < 0.05). They were primarily delayed mucosal healing and dry socket (Table 5).

**Table 5:** Frequency and nature of post-operative complications depending on the oral care of patients on biologics or immunosuppressants

	**All practitioners**	**Specialized dentist activity**^**c**^	**General dentist activity**	***P-value***	**All practitioners**	**Specialized dentist activity**^**c**^	**General dentist activity**	***P-value***
	**In patients under biologics**^**a**^				**In patients under immunosuppressants**^**b**^			
	***N* = 104 (%)**	***N* = 47 (%)**	***N* = 57 (%)**		***N* = 103 (%)**	***N* = 47 (%)**	***N* = 56 (%)**	
**Postoperative complications occurring after dental invasive procedures**								
No	91 *(87.50)*	37 *(78.72)*	54 *(94.74)*		84 *(81.55)*	33 *(70.21)*	51 *(91.07)*	
Yes	13 *(12.50)*	10 *(21.28)*	3 *(5.26)*	**0.01**	19 *(18.45)*	14 *(29.79)*	5 *(8.93)*	**0.007**
Endodontic procedures	0 *(0.00)*	0 *(0.00)*	0 *(0.00)*	-	0 *(0.00)*	0 *(0.00)*	0 *(0.00)*	-
Scaling and root planing	0 *(0.00)*	0 *(0.00)*	0 *(0.00)*	-	0 *(0.00)*	0 *(0.00)*	0 *(0.00)*	-
Procedures requiring mucoperiosteal flap rising with bone cutting/curetting	5 *(4.81)*	5 *(10.64)*	0 *(0.00)*	**0.01**	6 *(5.83)*	5 *(10.64)*	1* (1.79)*	0.05
Soft-tissue surgery	1 *(0.96)*	1 *(2.13)*	0 *(0.00)*	0.26	0 *(0.00)*	0 *(0.00)*	0 *(0.00)*	-
Dental extraction(s)	13 *(12.50)*	10 *(21.28)*	3 *(5.26)*	**0.01**	19 *(18.45)*	14 *(29.79)*	5 *(8.93)*	**0.007**
Implant(s) placement	2 *(1.92)*	2 *(4.26)*	0 *(0.00)*	0.11	2 *(1.94)*	2 *(4.26)*	0 *(0.00)*	0.11
**Type of postoperative complications after invasive dental procedures**								
Abscesses	2 *(1.92)*	2 *(4.26)*	0 *(0.00)*	0.11	3 *(2.91)*	2 *(4.26)*	1 *(1.79)*	0.45
Cellulitis	2 *(1.92)*	2 *(4.26)*	0 *(0.00)*	0.11	3 *(2.91)*	1 *(2.13)*	2 *(3.57)*	0.66
Dry socket	10 *(9.62)*	7 *(14.89)*	3 *(5.26)*	0.09	12 *(11.65)*	10 *(21.28)*	2 *(3.57)*	**0.005**
Osteitis	4 *(3.85)*	2 *(4.26)*	2 *(3.51)*	0.84	3 *(2.91)*	2 *(4.26)*	1 *(1.79)*	0.45
Delayed mucosal healing	12 *(11.54)*	10 *(21.28)*	2 *(3.51)*	**0.005**	15 *(14.56)*	12 *(25.53)*	3 *(5.36)*	**0.004**
Implant(s) osseointegration failure	0 *(0.00)*	0 *(0.00)*	0 *(0.00)*	-	1 *(0.97)*	1 *(2.13)*	0 *(0.00)*	0.27
Jaw-bone osteonecrosis	5 *(4.81)*	4 *(8.51)*	1 *(1.75)*	0.10	1 *(0.97)*	1 *(2.13)*	0 *(0.00)*	0.27

^a^Only practitioners who responded that they are concerned about the care of patients taking biologics (*n* = 104) were included in this analysis

^b^Only practitioners who responded that they were concerned about the care of patients taking immunosuppressants (*n* = 103) were included in this analysis

^c^Specialized dentist activity: oral surgery, periodontology, endodontics, pediatric dentistry

### Evaluation of the relationship between the management of medical treatment and oral care protocols, and the complications of invasive oral procedures

The continuation of bDMARDs treatment correlated with the occurrence of complications after raising of a mucoperiosteal flap with bone resection/ curetting (*p* = 0.04), and dry socket (*p* = 0.05) in the SD group (Table 6). For conventional DMARDs/ ISs, the continuation of the treatment correlated with more complications after dental extraction(s) (*p* = 0.02) or raising of mucoperiosteal flaps with bone resection/ curetting (*p* = 0.02) in the SD group (Table 7). The continuation of conventional DMARDs/ ISs correlated with delayed mucosal healing (*p* = 0.01) and dry sockets (*p* = 0.05) in the SD group (Table 8).

**Table 6:** Management of medical treatment and the complications of invasive procedures for patients taking biologics

	**All practitioners** ***N* = 104 (%)**^**a**^			**Specialized dentist activity**^**b**^ ***N* = 47 (%)**			**General dentist activity** ***N* = 5 7 (%)**		
	**Complications after mucoperiosteal flap rising with bone resection/curetting**								
	**No**	**Yes**	***p-value***	**No**	**Yes**	***p-value***	**No**	**Yes**	***p-value***
**Preoperative management of the medical treatment**									
No modification	35 *(33.65)*	4 *(3.85)*	**0.04**	14 *(29.78)*	4 *(8.51)*	**0.04**	21 *(36.84)*	0 *(0.00)*	-
Discontinuation (different protocols)	64 *(61.53)*	1 *(0.96)*		28 *(59.57)*	1 *(2.13)*		36 *(63.16)*	0 *(0.00)*	
**Pre, per, and postoperative dental-care protocol**									
***Antiseptic mouthwash***									
Never	10 *(9.62)*	0 *(0.00)*	0.45	5 *(10.64)*	0 *(0.00)*	0.41	5* (8.77)*	0 *(0.00)*	-
Ever (pre, per, and/or postoperative)	89 *(85.57)*	5 *(4.81)*		37 *(78.72)*	5 *(10.64)*		52 *(91.23)*	0 *(0.00)*	
***Antibiotics***									
Never	6 *(5.77)*	0 *(0.00)*	0.84	1 *(2.13)*	0 *(0.00)*	0.89	5* (8.77)*	0 *(0.00)*	-
Exclusively single dose preoperatively	16 *(15.38)*	1 *(0.96)*		6 *(12.77)*	1 *(2.13)*		10 *(17.54)*	0 *(0.00)*	
Antibiotic coverage/both single dose preoperatively and antibiotic coverage	77 *(74.04)*	4* (3.85)*		35* (74.47)*	*4 (8.51)*		42 *(73.69)*	0 *(0.00)*	
	**Dry socket**								
	**No**	**Yes**	***p-value***	**No**	**Yes**	***p-value***	**No**	**Yes**	***p-value***
**Preoperative management of the medical treatment**									
No modification	33 *(31.73)*	6 *(5.76)*	0.12	13 *(27.66)*	5 *(10.64)*	0.05	20 *(35.09)*	1 *(1.75)*	0.89
Discontinuation (different protocols)	61 *(58.65)*	4 *(3.85)*		27 *(57.44)*	2 *(4.26)*		34 *(59.65)*	2 *(3.51)*	
**Pre, per, and postoperative dental-care protocol**									
***Antiseptic mouthwash***									
Never	10* (9.62)*	0 *(0.00)*	0.27	5 *(10.64)*	0 *(0.00)*	0.32	5* (8.77)*	0 *(0.00)*	0.58
Ever (pre, per, and/or postoperative)	84 *(80.76)*	10* (9.62)*		35* (74.47)*	7* (14.89)*		49 *(85.97)*	3* (5.26)*	
***Antibiotics***									
Never	6 *(5.77)*	0 *(0.00)*	0.37	1 *(2.13)*	0 *(0.00)*	0.07	5* (8.77)*	0 *(0.00)*	0.56
Exclusively single dose preoperatively	14 *(13.46)*	3* (2.88)*		4 *(8.51)*	3* (6.39)*		10 *(17.54)*	0* (0.00)*	
Antibiotic coverage/both single dose preoperatively and antibiotic coverage	74 *(71.15)*	7 (6.73)		35* (74.47)*	4 *(8.51)*		39 *(68.43)*	3 *(5.26)*	

^a^Only practitioners who responded that they are concerned about the care of patients taking biologics (*n* = 104) were included in this analysis

^b^Specialized activity: oral surgery, periodontology, endodontics, pediatric dentistry

**Table 7:** Management of medical treatment, infection risk prevention procedures, and reporting of complications of invasive dental procedures for patients taking immunosuppressants

	**All practitioners** ***N* = 103 (%)**^**b**^			**Specialized dentist activity**^**a**^ ***N* = 47 (%)**			**General dentist activity** ***N* = 56 (%)**		
	**Complications after dental extraction(s)**								
	**No**	**Yes**	***p-value***	**No**	**Yes**	***p-value***	**No**	**Yes**	***p-value***
**Preoperative management of the medical treatment**									
No modification	39 *(37.86)*	13 *(12.62)*	0.08	14 *(29.79)*	11 *(23.40)*	**0.02**	25 *(44.64)*	2 *(3.57)*	0.70
Discontinuation (different protocols)	45 *(43.69)*	6 *(5.83)*		19 *(40.43)*	3 *(6.38)*		26 *(46.43)*	3 *(5.36)*	
**Pre, per, and postoperative dental-care protocol**									
***Antiseptic mouthwash***									
Never	15 *(14.56)*	0 *(0.00)*	**0.04**	7 *(14.89)*	0 *(0.00)*	0.06	*8 (14.29)*	0 *(0.00)*	0.33
Ever (pre, per, and/or postoperative)	69 *(67.00)*	19 *(18.45)*		26 *(55.32)*	14 *(29.79)*		43 *(76.78)*	*5 (8.93)*	
***Antibiotics***									
Never	3 *(2.91)*	0 *(0.00)*	0.34	1 *(2.13)*	0 *(0.00)*	0.33	2 *(3.57)*	0 *(0.00)*	0.88
Exclusively a single dose preoperatively	12 *(11.65)*	5 *(4.85)*		4 *(8.51)*	4 *(8.51)*		*8 (14.29)*	1 *(1.79)*	
Exclusively antibiotic coverage/both single dose and antibiotic coverage	69 *(67.00)*	14 *(13.59)*		28 (59.57)	10 *(21.28)*		41 *(73.21)*	4 *(7.14)*	
	**Complications after mucoperiosteal flap rising with bone resection/curetting**								
	**No**	**Yes**	***p-value***	**No**	**Yes**	***p-value***	**No**	**Yes**	***p-value***
**Preoperative management of the medical treatment**									
No modification	46 *(44.66)*	6 *(5.83)*	**0.01**	20 *(42.55)*	5 *(10.64)*	**0.02**	26 *(46.42)*	1 *(1.79)*	0.29
Discontinuation (different protocols)	51 *(49.51)*	0 *(0.00)*		22 *(46.81)*	0 *(0.00)*		29* (51.79)*	0 *(0.00)*	
**Pre, per, and postoperative dental-care protocol**									
***Antiseptic mouthwash***									
Never	15 *(14.56)*	0 *(0.00)*	0.29	7 *(14.89)*	0 *(0.00)*	0.32	*8 (14.29)*	0 *(0.00)*	0.68
Ever (pre, per, and/or postoperative)	82* (79.61)*	6 *(5.83)*		35 *(74.47)*	5 *(10.64)*		47 *(83.92)*	1 *(1.79)*	
***Antibiotics***									
Never	3 *(2.91)*	0 *(0.00)*	0.90	1 *(2.13)*	0 *(0.00)*	0.92	2 *(3.57)*	0 *(0.00)*	0.88
Exclusively a single dose preoperatively	16 *(15.54)*	1 *(0.97)*		7 *(14.89)*	1 *(2.13)*		9 *(16.07)*	0 *(0.00)*	
Exclusively antibiotic coverage/both single dose and antibiotic coverage	78 *(75.73)*	5 *(4.85)*		34* (72.34)*	4 *(8.51)*		44* (78.57)*	1 *(1.79)*	

^a^Specialized activity: oral surgery, periodontics, endodontics, pediatric dentistry

^b^Only practitioners who responded that they are concerned about the care of patients taking immunosuppressants (*n* = 103) were included in this analysis

**Table 8:** Management of medical treatment, infection risk prevention procedures, and the type of complication of invasive procedures for patients taking immunosuppressants

	**All practitioners** ***N* = 103 (%)**^**b**^			**Specialized dentist activity**^**a**^ ***N* = 47 (%)**			**General dentist activity** ***N* = 56 (%)**		
	**Dry socket**								
	**No**	**Yes**	***p-value***	**No**	**Yes**	***p-value***	**No**	**Yes**	***p-value***
**Preoperative management of the medical treatment**									
No modification	43 *(41.75)*	9 *(8.74)*	0.07	17* (36.17)*	8 *(17.02)*	0.05	26 *(46.42)*	1 *(1.79)*	0.95
Discontinuation (different protocols)	48 *(46.60)*	3 *(2.91)*		20 *(42.55)*	2* (4.26)*		28 *(50.00)*	1 *(1.79)*	
**Pre, per, and post operative dental-care protocol**									
***Antiseptic mouthwash***									
Never	15 *(14.56)*	0 *(0.00)*	0.12	7 *(14.89)*	0 *(0.00)*	0.13	*8 (14.29)*	0 *(0.00)*	0.55
Ever (pre, per, and/or postoperative)	76 *(73.79)*	12 *(11.65)*		30* (63.83)*	10* (21.28)*		46 *(82.14)*	2 *(3.57)*	
***Antibiotics***									
Never	3 *(2.91)*	0 *(0.00)*	0.21	1 *(2.13)*	0 *(0.00)*	0.08	2 *(3.57)*	0 *(0.00)*	0.77
Exclusively a single dose preoperatively	13 *(12.62)*	4 (3.88)		4 *(8.51)*	4 *(8.51)*		9 *(16.07)*	0 *(0.00)*	
Exclusively antibiotic coverage/both single dose and antibiotic coverage	75* (72.82)*	8 *(7.77)*		32 *(68.09)*	6 *(12.76)*		43* (76.79)*	2 *(3.57)*	
	**Delayed mucosal healing**								
	**No**	**Yes**	***p-value***	**No**	**Yes**	***p-value***	**No**	**Yes**	***p-value***
**Preoperative management of the medical treatment**									
No modification	40 *(38.84)*	12 *(11.65)*	**0.01**	15* (31.91)*	10* (21.28)*	**0.01**	25 *(44.64)*	2 *(3.57)*	0.51
Discontinuation (different protocols)	48 *(46.60)*	3 *(2.91)*		20 *(42.55)*	2* (4.26)*		28 *(50.00)*	1 *(1.79)*	
**Pre, per, and post operative dental-care protocol**									
***Antiseptic mouthwash***									
Never	15 *(14.56)*	0 *(0.00)*	0.08	7 *(14.89)*	0 *(0.00)*	0.09	*8 (14.29)*	0 *(0.00)*	0.46
Ever (pre, per, and/or postoperative)	73 *(70.88)*	15 *(14.56)*		28 *(59.57)*	12 *(25.54)*		45 *(80.35)*	3 *(5.36)*	
***Antibiotics***									
Never	3 *(2.91)*	0 *(0.00)*	0.72	1 *(2.13)*	0 *(0.00)*	0.60	2 *(3.57)*	0 *(0.00)*	0.67
Exclusively a single dose preoperatively	14 *(13.59)*	3 *(2.91)*		5 *(10.64)*	3 *(6.38)*		9 *(16.07)*	0 *(0.00)*	
Exclusively antibiotic coverage/both single dose and antibiotic coverage	71 *(68.94)*	12 *(11.65)*		29* (61.70)*	9 *(19.15)*		42 *(75.00)*	3 *(5.36)*	

^a^Specialized activity: oral surgery, periodontics, endodontics, pediatric dentistry

^b^Only practitioners who responded that they are concerned about the care of patients taking immunosuppressants (*n* = 103) were included in this analysis

## Discussion

This study is among the largest to compare the practice of French dentists with existing national recommendations on the management of oral care in patients with IMIDs treated with chronic medications. Most of the participants reported having experience in the management of patients with IRD and IBD than for vasculitis, consistent with the prevalence of these diseases. They also declared being aware of the professional recommendations. Overall, complications were more frequently reported by SDs. It can be explained by the fact that they used (i) to treat patients with more severe and unstable forms of the disease with the continuation of immunosuppressive therapy and (ii) to perform extensive surgical procedures with bone cutting. Of note, no complications were reported after procedures such as scaling, which is regularly performed during the patient’s annual follow-up. This result is highly reassuring.

In terms of the management of the risk of infection, most dentists applied the recommended procedures for prevention of infection by prescribing antiseptics and antibiotics until mucosal healing [6]. Despite these precautions, a notable percentage of infections or delayed healing was reported. These complications were more frequent when the procedure was invasive and extensive, and they correlated with the continuation of treatment.

Of note, the current joint American College of Rheumatology/American Association of Hip and Knee Surgeons recommendations (Grade B recommendations) for the perioperative management of patients with RA or systemic lupus erythematosus in orthopedic surgery concludes that (1) conventional DMARDs should be continued for orthopedic surgery and (2) bDMARDs should be temporarily suspended perioperatively (depending on the molecule). bDMARDs should be resumed approximately two weeks after surgery [11, 12]. The paucity of literature on the risk of postoperative infection or the risk of a disease flare-up is emphasized, especially for bDMARDs, and particularly for oral surgery cases.

To date, literature on that topic in oral surgery is scarce. In a recent Cochrane systematic review on the prevention of complications after tooth extraction, none of the included studies evaluated tooth extraction in immunocompromised patients [13]. It is however known that there is a correlation between the invasiveness of oral surgery and the incidence and severity of postoperative complications in the general population. For example, in a retrospective study of 3,900 patients, complications occurred more frequently after the extraction of impacted mandibular or third maxillary molars and bone surgery. These complications were mainly alveolar osteitis, infection, or prolonged swelling and occurred more frequently in the presence of other risk factors of infection [14]. Here, we conducted a declarative questionnaire to carry out a practice survey, but our objective was not to investigate risk factors for infections following invasive care. If this had been the case, assessing the use of bisphosphonates, which are widely prescribed for patients with rheumatoid arthritis, would have been an essential point to note because they increase the risk of postoperative complications [15]. Indeed, cases of osteonecrosis of the jaw in the absence of antiresorptive or antiangiogenic drugs have been described in patients with rheumatoid arthritis or idiopathic arthritis treated with prednisolone, methotrexate, or bDMARDs (adalimumab) [16, 17]. In contrast without our study, only 18% of the respondents in a recent Japanese survey that included 206 dentists [13] asked the prescribing physician to request temporary discontinuation of conventional DMARDs, bDMARDS, ISs, or corticoids. These discrepancies with our results could be explained by different recommendations between countries.

One limitation of our study is the representativity of the French dentist population. The number of participants in the present study was small with respect to the population of dentists in France (44,000 according to the National Council of the Order of Dental Surgeons [18]). The participation rate was estimated at approximately 11%. It is consistent with previous professional practice studies, for example, in the perioperative oral care management of patients on anticoagulants, a condition which concerns more patients and practitioners than the subject of our study [19]. A disparity was observed in the response rate of dentists according to region, consistent with the current demography of dentists in the French territory [20]. Moreover, an over-representation of SDs was observed, which can be explained by a recruitment bias due to (i) the way the survey was distributed, (ii) a particular interest in the subject of the survey, and (iii) better adherence to medical scientific studies.

The second major limitation is due to the method of data collection. The use of a self-administered questionnaire is per se associated with various biases. Measurement bias was limited by having the questionnaire tested by a panel of dentists, which were distributed via numerous societies and colleges. Recall and subjective biases were not excluded. For example, a significant difference between the percentages of practitioners who did not know the names of bDMARDs versus ISs or cDMARDs was observed. However, it may be explained by the fact that dentists are more familiar with ISs or cDMARDs, which are more widely prescribed and have been prescribed for longer. Nevertheless, even though a self-administered questionnaire may not be as well completed as a questionnaire administered in face-to-face interviews, it does allow for the inclusion of a greater number of participants.

## Conclusion

This survey shows that most SDs and GDs perform a wide range of oral procedures on patients using bDMARDS, conventional DMARDs, or ISs. They declared being compliant with the recommendations for oral invasive care of treating patients under antibiotic coverage and antiseptic mouthwashes. Due to their education and expertise, SDs more frequently performed invasive and complex treatments (multiple tooth extraction, surgery with open flap access and bone cutting). They thus reported having more patients with postoperative complications, despite having greater knowledge regarding recommendations for the management of such patients. Complications appear to mainly occur after highly invasive procedures, possibly when ISs are not suspended. Finally, certain common procedures, such as scaling and root planing appear to be safe. However, adapted dental practice guidelines are needed to standardize the management of patients on bDMARDS, conventional DMARDs, or ISs. Real-life data obtained by conducting sufficiently powerful prospective studies are needed as the present study was only a declarative survey.

### Supplementary Information

**Additional file 1: Supplemental Figure 1.** Training of general and specialist dentists in France.

**Additional file 2: Supplemental Table 1.** Management of medical treatment and oral care of patients on biologics or immunosuppressants.

## Data Availability

The datasets used during the current study available from the corresponding author on reasonable request.
