# Supplementary material for: Management of dental care of patients on immunosuppressive drugs for chronic immune-related inflammatory diseases: a survey of French dentists’ practices
Source: BMC Oral Health. 2023 Aug 9;23:545. doi: 10.1186/s12903-023-03258-7 (PMC10411020; doi:10.1186/s12903-023-03258-7)
Supplement: Supplementary file 1 — Additional file 1: Supplemental Figure 1. Training of general and specialist dentists in France. [file 12903_2023_3258_MOESM1_ESM.pptx]

## Slide 1
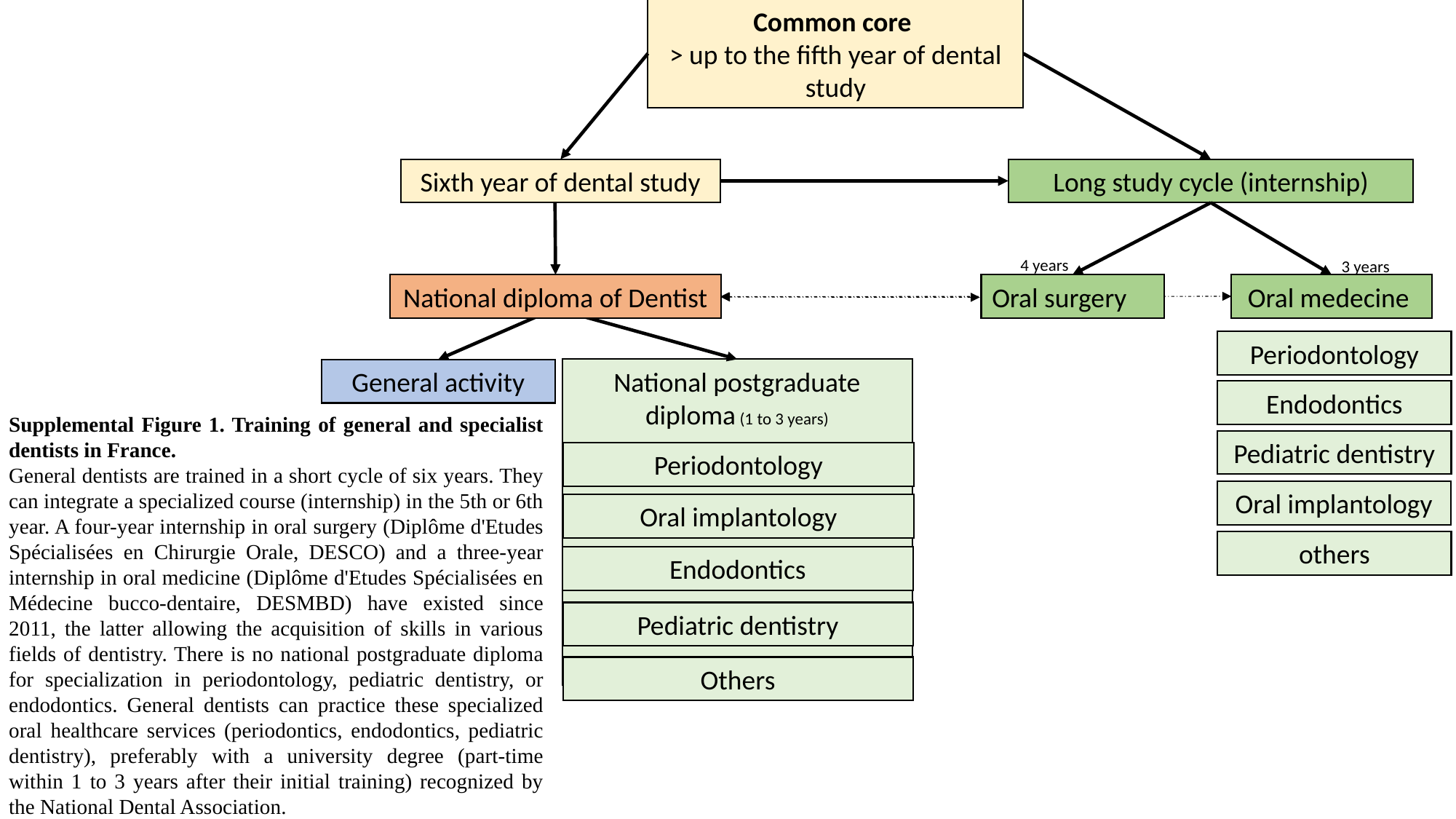

Common core
> up to the fifth year of dental study
Sixth year of dental study
Long study cycle (internship)
4 years
3 years
National diploma of Dentist
Oral surgery
Oral medecine
Periodontology
National postgraduate diploma (1 to 3 years)
General activity
Endodontics
Supplemental Figure 1. Training of general and specialist dentists in France.
General dentists are trained in a short cycle of six years. They can integrate a specialized course (internship) in the 5th or 6th year. A four-year internship in oral surgery (Diplôme d'Etudes Spécialisées en Chirurgie Orale, DESCO) and a three-year internship in oral medicine (Diplôme d'Etudes Spécialisées en Médecine bucco-dentaire, DESMBD) have existed since 2011, the latter allowing the acquisition of skills in various fields of dentistry. There is no national postgraduate diploma for specialization in periodontology, pediatric dentistry, or endodontics. General dentists can practice these specialized oral healthcare services (periodontics, endodontics, pediatric dentistry), preferably with a university degree (part-time within 1 to 3 years after their initial training) recognized by the National Dental Association.
Pediatric dentistry
Periodontology
Oral implantology
Oral implantology
others
Endodontics
Pediatric dentistry
Others
