# Supplementary material for: Management of dental care of patients on immunosuppressive drugs for chronic immune-related inflammatory diseases: a survey of French dentists’ practices
Source: BMC Oral Health. 2023 Aug 9;23:545. doi: 10.1186/s12903-023-03258-7 (PMC10411020; doi:10.1186/s12903-023-03258-7)
Supplement: Supplementary file 2 — Additional file 2: Supplemental Table 1. Management of medical treatment and oral care of patients on biologics or immunosuppressants. [file 12903_2023_3258_MOESM2_ESM.pdf]

Supplemental Table 1. Management of medical treatment and oral care of patients on biologics or immunosuppressants.

	All practitioners	Specialized dentist activity *	General dentist activity	*p-value*	All practitioners	Specialized dentist activity*	General dentist activity	*p-value*
	**In patients under biologics**				**In patients under immunosuppressants**			
	**N = 107 (%)**	**N = 49 (%)**	**N = 58 (%)**		**N = 107 (%)**	**N = 49 (%)**	**N = 58 (%)**	
**Nature of the expected risks**								
Infectious risk	105 *(98.13)*	49 *(100.00)*	56 *(96.55)*	0.18	106 *(99.07)*	49 *(100.00)*	57 *(98.28)*	0.35
Delayed healing	98 *(91.59)*	46 *(93.88)*	52 *(89.66)*	0.43	90 *(84.11)*	40 *(81.63)*	50 *(86.21)*	0.51
Hemorrhagic risk	20 *(18.69)*	10 *(20.41)*	10 *(17.24)*	0.67	13 *(12.15)*	5 *(10.20)*	8 *(13.79)*	0.57
	**N = 104 (%)[a]**	**N = 47 (%)[a]**	**N = 57 (%)[a]**		**N = 103 (%)[b]**	**N = 47 (%)[b]**	**N = 56 (%)[b]**	
**Blood tests prescribed preoperatively**								
No	80 *(76.92)*	32 *(68.09)*	48 *(84.21)*		74 *(71.84)*	31 *(65.96)*	43 *(76.79)*	
Yes	24 *(23.08)*	15 *(31.91)*	9 *(15.79)*	0.05	29 *(28.16)*	16 *(34.04)*	13 *(23.21)*	0.22
Blood cell count	23 *(95.83)*	15 *(100.00)*	8 *(88.89)*	0.18	28 *(96.55)*	16 *(100.00)*	12 *(92.31)*	0.25
Platelet cell count	17 *(70.83)*	11 *(73.33)*	6 *(66.67)*	0.72	14 *(48.28)*	8 *(50.00)*	6 *(46.15)*	0.83
**Preoperative management of the medical treatment**								
No modification	65 *(62.50)*	29 *(61.70)*	36 *(63.16)*	0.87	51 *(49.51)*	22 *(46.81)*	29 *(51.79)*	0.61
Discontinuation ≤ 1 month	8 *(7.69)*	4 *(8.51)*	4 *(7.02)*	0.77	5 *(4.85)*	3 *(6.38)*	2 *(3.57)*	0.50
Discontinuation > 1 month	4 *(3.85)*	2 *(4.26)*	2 *(3.51)*	0.84	1 *(0.97)*	1 *(2.13)*	0 *(0.00)*	0.27
Duration of discontinuation depending on the type of drug	3 *(2.88)*	2 *(4.26)*	1 *(1.75)*	0.55	1 *(0.97)*	1 *(2.13)*	0 *(0.00)*	0.27
Decision after consultation with the prescribing physicians	84 *(80.77)*	41 *(87.23)*	43 *(75.44)*	0.12	79 *(76.70)*	38 *(80.85)*	41 *(73.21)*	0.36
**Pre, per, and post- operative dental-care protocol**								
Antiseptic mouthwash								
Preoperative	74 *(71.15)*	33 *(70.21)*	41 *(71.93)*	0.84	69 *(66.99)*	31 *(65.96)*	38 *(67.86)*	0.83
Perioperative	52 *(50.00)*	25 *(53.19)*	27 *(47.37)*	0.55	55 *(53.40)*	27 (57.45)	28 *(50.00)*	0.45
Postoperative	79 *(75.96)*	37 *(78.72)*	42 *(73.68)*	0.54	74 *(71.84)*	32 *(68.09)*	42 *(75.00)*	0.43
Antibiotics								
Preoperative (single dose, flash)	49 *(47.12)*	20 *(42.55)*	29 *(50.88)*	0.39	45 *(43.69)*	20 *(42.55)*	25 *(44.64)*	0.83
Preoperative and postoperative (antibiotic coverage until mucosal healing)	81 *(77.88)*	39 *(82.98)*	42 *(73.68)*	0.25	83 *(80.58)*	38 *(80.85)*	45 *(80.36)*	0.95

* Specialized dentist activity: oral surgery, periodontology, endodontics, pediatric dentistry

[a] Only practitioners who responded that they are concerned about the care of patients taking biologics (n = 104) were included in this analysis.

[b] Only practitioners who responded that they were concerned about the care of patients taking immunosuppressants (n = 103) were included in this analysis.
